# From erasure to opportunity: a qualitative study of the experiences of transgender men around pregnancy and recommendations for providers

**DOI:** 10.1186/s12884-017-1491-5

**Published:** 2017-11-08

**Authors:** Alexis Hoffkling, Juno Obedin-Maliver, Jae Sevelius

**Affiliations:** 1 School of Medicine, University of California San Francisco, 2926 Otis St., Berkeley, CA 94703 USA; 2 Department of Obstetrics, Gynecology & Reproductive Sciences, University of California, San Francisco, 550 16th Street, San Francisco, CA 94158 USA; 3 Department of General Internal Medicine, San Francisco Department of Veterans Affairs, 4150 Clement Street, Building 18, 111A1, San Francisco, CA 94142 USA; 4 Center for Excellence in Transgender Health, University of California San Francisco, 550 16th Street, San Francisco, CA 94158 USA

**Keywords:** Transgender, Pregnancy, Stigma, Transsexual, Female-to-male, Reproduction, Lactation

## Abstract

**Background:**

Some transgender men retain their uterus, get pregnant, and give birth. However, societal attitudes about gender have erected barriers to openly being pregnant and giving birth as a transgender man. Little research exists regarding transgender men’s reproductive needs. Anecdotal observations suggest that social change and increasing empowerment of transgender men may result in increasing frequency and openness about pregnancy and birth. Specific needs around conception, pregnancy, and newborn care may arise from transphobia, exogenous testosterone exposure, or from having had (or desiring) gender-affirming surgery. We undertook a qualitative study to understand the needs of transgender men who had given birth.

**Methods:**

We interviewed 10 transgender men who had been recruited for a recently published online cross-sectional survey of individuals (n = 41). Subjects had given birth while identifying as male. Interviews were recorded, transcribed, and systematically coded. Analysis used a priori and emergent codes to identify central themes and develop a framework for understanding participant experiences.

**Results:**

Participants reported diverse experiences and values on issues including prioritization and sequencing of transition versus reproduction, empowerment in healthcare, desire for external affirmation of their gender and/or pregnancy, access to social supports, and degree of outness as male, transgender, or pregnant. We identified structural barriers that disempowered participants and describe healthcare components that felt safe and empowering. We describe how patients’ strategies, and providers’ behaviors, affected empowerment. Anticipatory guidance from providers was central in promoting security and empowerment for these individuals as patients.

**Conclusions:**

Recognizing diverse experiences has implications in supporting future patients through promoting patient-centered care and increasing the experiential legibility. Institutional erasure creates barriers to transgender men getting routine perinatal care. Identifying this erasure helps shape recommendations for how providers and clinics can provide appropriate care. Specific information regarding reproduction can be helpful to patients. We provide recommendations for providers’ anticipatory guidance during the pre-transition, pre-conception, prenatal, and postpartum periods. Ways to support and bring visibility to the experience of transgender men are identified. Improving clinical visibility and affirming gender will likely enhance patient experience and may support patient-centered perinatal healthcare services.

**Electronic supplementary material:**

The online version of this article (10.1186/s12884-017-1491-5) contains supplementary material, which is available to authorized users.

## Background

Transgender men are individuals who identify as men but were assigned female sex at birth (Table 1 for definitions of terms). Many transgender men, and other gender nonconforming individuals, retain their ovaries and uterus as well as the capacity to become pregnant. Indeed, some of these men are becoming pregnant and giving birth [1]. While some research and guidelines have been published regarding the gynecologic care of transgender men [2–4], there has been little investigation into the fertility and pregnancy-related needs of this population [5]. Transgender people, as a group, have faced disempowerment via stigma, discrimination, and bias, as well as experiencing numerous health disparities [6]. As a historically underserved group, transgender people warrant attention from researchers and providers in terms of identifying and addressing their particular barriers and needs [2]. Kabeer characterizes empowerment as movement away from disempowerment, “*…empowerment is about change, it refers to the expansion in people’s ability to make strategic life choices, in a context where this ability was previously denied to them*” [7]. In addition to the many forms of disempowerment faced by most transgender individuals, there are specific restrictions on transgender individuals’ reproductive choices. Some jurisdictions have required that individuals be sterilized in order to attain legal recognition of their gender [8]. In this context, the very act of choosing to have children as a transgender man is an act of empowerment. Navigating the details of this process involves countless further strategic life choices, many of which occur in the face of disempowering factors.

**Table 1:** Definitions of terms

Transgender (‘trans’): Having a gender identity that differs from the sex assigned at birth (e.g. identifying as male, and having been assigned ‘female’ at birth.)
Cisgender: Having a gender identity that matches the sex assigned at birth (e.g. identifying as female, and having been assigned ‘female’ at birth.)
Cissexism/Transphobia: the values, attitudes, and actions that value cisgender individuals’ lives and experiences over those of transgender individuals. Examples include: seeing being transgender as bad; violence against individuals who are (perceived as) transgender; making things more difficult for transgender people than cisgender people, etc.

Despite societal gains toward the empowerment of transgender individuals, there remains ongoing violence against this population. Violence here is conceived broadly, encompassing economic, legal, medical, psychological and physical violence. Outside of healthcare settings, stigma disempowers transgender individuals by producing barriers to economic opportunity, exposure to physical violence, and chronic stressors [9]. Within healthcare settings, stigma leads to inadequate information on the part of providers, as well as individual mistreatment of patients [9]. These, in turn, can lead transgender men to avoid seeking care or avoid disclosing medically relevant information [6]. This violence and disempowerment has an ongoing cost for the health and wellbeing of transgender individuals [6, 10, 11].

This population’s health disparities and increased barriers to care are partly perpetuated by structural forces within healthcare. In addition to widespread stigma, these structural forces include inadequate research and insufficient educational and institutional attention to the needs of this population (collectively termed erasure) [2].

Erasure, transphobia, and violence likely produce barriers to appropriate reproductive care for transgender people, though how this plays out is not well understood [5]. Additionally, transgender individuals likely have specific needs pertaining to fertility, conception, pregnancy, delivery, and the postpartum period compared to the general population. These needs could arise from the biomedical effects of prior or intended exogenous hormone use or gender-affirming surgeries [5]. Cultural and structural features of our society and institutions likely produce unique needs for this population, including anti-transgender stigma, strongly gendered norms around pregnancy, institutional structures that do not recognize the possibility of a transgender man becoming pregnant, and lack of research and available information for providers or patients [10, 12]. Psychosocially, transgender men may have specific needs arising from their relationship with their gender identity, body dysphoria, or others’ perceptions of their pregnant body [5, 12].

We aim to identify some of the needs of transgender men in the family planning process and during the peripartum period, as well as the ways they have achieved empowerment, opportunities for supporting their further empowerment, and priorities for further investigation through a systematic qualitative study.

## Methods

### Theoretical framework

This study used a grounded theory approach [13] to explore the experiences of transmasculine individuals’ experience with pregnancy through focused, semi-structured interviews with 10 participants. Because little is known about this topic from research or theory, grounded theory allowed inductive pattern finding through structured qualitative data collection and analysis.

### Participants

Participants were recruited for interviews from a pool of prior participants of an online convenience sampled survey of transmasculine individuals who had given birth, conducted in 2013 [1, 14]. Inclusion criteria were 18 years or older, self-identification as male before pregnancy, pregnant within the last 10 years, and the ability to complete the survey in English. Eligibility criteria did not require participants to have undergone any type of medical (e.g., testosterone use) or surgical (e.g., bilateral mastectomy) transition intervention [1]. After completing the survey, participants could opt for future contact for further studies. Of the 41 participants in the original survey, 23 gave follow-up contact information and were invited to participate in subsequent interviews. Thirteen responded, of which one was deemed ineligible. Interviews were conducted, over a 2-month period, until theoretical saturation was reached at 10 interviews. Interview order was based on response speed.

### Interviews

Interviews were conducted online, using the video remote-conferencing software program BlueJeans for eight interviews and two were conducting using audio only. The lead author (AH) conducted all interviews. Participants were emailed a consent form 2–4 days before the interview, which was reviewed aloud at the beginning of the interview to obtain verbal consent. Interviews followed a semi-structured format. Closed-ended demographic questions were also administered at interview end. Participants were compensated with a $35 Amazon gift card or PayPal payment, according to participant preference. Participants could choose to have their contact information destroyed or retained to facilitate distribution of published study results.

### Analysis

Interviews were recorded, professionally transcribed, and coded using Dedoose qualitative analysis software. Two transcripts, selected purposively for diversity of content, were coded using a priori codes, and emergent codes were identified and applied. Emergent codes were reviewed by three researchers to produce the final code set, and then all transcripts, including the initial transcripts used for code-development, were (re)coded using the final code set. Two of these transcripts were fully coded by a second researcher, and reviewed by two more, for validation of coding reliability. No major differences of interpretation arose, indicating a high level of coding reliability.

Because of the small sample size in this study, the fact that not all interviews covered all the topics, and the likely selection bias due to convenience sampling, we chose not to present any quantitative data such as frequency counts.

## Results

### Diversity

A central finding was the wide variation within the population of transgender men giving birth, along many axes of difference, including identity, reproductive intent, fecundity and gamete source, need for affirmation of identity and pregnancy, social support, degree of outness, and priorities and sequencing of transition and reproduction.

#### Identity

Inclusion criteria for this study required patients to identify as male at the time of their pregnancy. Participants in this study described themselves, variously, as ‘male,’ ‘man,’ ‘female-to-male,’ ‘transman,’ ‘trans man,’ ‘transgender man,’ ‘transmasculine,’ ‘nonbinary,’ and ‘on the transmasculine spectrum.’ Some participants had a clear preference, and others were comfortable with a variety of terms.

#### Reproductive intent

Participants described their pregnancies as, variously, strongly desired, necessary to build a family, or unintended. Some unintended pregnancies occurred after male identification but prior to any medical or surgical transition, “*I first started questioning my gender at about age 19… when I was 20 I talked to a doctor about* [testosterone]*, and was waiting to get that started* [when] *I accidentally got pregnant*.” Some chose pregnancy as a tolerable means to become a parent, “*but if I want to reproduce, that is the only way I can do it. So I agreed this - if I could do it another way, I would maybe do it another way, but I don’t have the option*,” while some enthusiastically desired pregnancy, “*I always knew that I wanted to have kids, and that I would be giving birth to my own kids*.” No participants reported serious consideration of terminating their pregnancy or of seeking healthcare related to elective termination.

#### Fecundity and gamete sources

There was variable fecundity; participants had had one to four pregnancies, with one to three live births. Participants conceived using sperm from committed partners, sexual partners with whom they had no intent to have a long-term relationship, known donors, and anonymous donors. All participants conceived using their own oocytes.

#### Access to social support

Participants’ social support ranged from robust to minimal and tenuous. For some, their pregnancy was a very isolating experience: “*I just lost everybody.*” While others found abundant support and affirmation from family, friends, and strangers:

“*In the queer community and the leather community… I had an overwhelmingly positive reaction… When it was really obvious that I was a pregnant tranny, I actually received a lot of positive love and affection from queer strangers…and I actually had strangers stop and ask if they could hug me and thought that it was beautiful.*”

Some participants directly cited their supportive communities as a source of resilience against the challenges they faced,

“*If I hadn’t had positive reactions* [from family] *in the very beginning of my transition… I would have been far more self-questioning, and less strong in standing up to* [others] *who wanted to tell me something that wasn’t true.*”

A particular source of support for several participants was the Facebook group “Birthing and Breastfeeding Trans People and Allies” (https://www.facebook.com/groups/449750635045499/ accessed April 2015). Several participants reported that pregnancy and parenting support organizations for gay, lesbian, and bisexual people were ill-equipped to support transgender parents.

#### Need for identity and pregnancy affirmation

Need for affirmation of gender identity was also highly variable. For some, being seen and treated as male – with consistent use of male names and pronouns – was critical to their sense of emotional safety and wellbeing. Others were minimally bothered by being misgendered. Similarly, for some, it was important that they be seen as pregnant. Others did not want their pregnancy known or acknowledged by anyone other than their close loved ones and medical providers.

“*I just didn’t like leaving the house at all because I knew that I was going to be read as pregnant female, and it just ugh. After I’d worked so hard the past couple of years to get* [people to see me as male].”

“[I wanted] *support from my community… so I told my coworkers and my synagogue* [that I was pregnant]. *I wrote an email… and it was really nice, how ridiculously excited they were for me.*”

#### Degree of outness

Visibility and ‘outness’ had to be considered in two domains. The choice of whether and how to be ‘out’, or visible as pregnant and/or transgender, played out in complex ways for the participants. Participants described a mix of strategies in navigating degree of outness, and most of the participants employed several of these strategies, varying by setting, whom they were with, and time during the pregnancy. The three most common strategies were (1) passing as a cisgender woman (i.e., one who identifies as a woman and was assigned female sex), (2) going stealth, and (3) being out and visible.

Strategy 1, passing as a cisgender woman (acting so as to incline others to think one is a cisgender woman), increased external affirmation of the pregnancy, but decreased external affirmation of male gender, as reported in the original online survey preceding these interviews [1]. This strategy increased some participants’ feelings of safety, and decreased their exposure to transphobic violence,

“[I was] *intentionally trying to be inconspicuous and fly below the radar. I wanted to be able to present as male, but I made that decision* [to present as female] *at that time because I was afraid.*”

At times it appeared that this strategy came at the expense of increasing dysphoria due to passing as a gender that does not align with their sense of self.

Strategy 2, going stealth (acting so as to incline others to think one is a cisgender male), increased external affirmation of gender and decreased exposure to transphobic violence, but also decreased external affirmation of the pregnancy. By not being visible as pregnant, some benefits were missed, including social support, physical assistance, and external affirmation. Those who pass as cisgender male report being consistently “*perceived as a fat man and never as a pregnant woman.*” It sometimes surprised participants how invisible their pregnancies were:

“*People could not process my masculine appearance with pregnancy… How can* [this cashier] *think that I’m male when I’m eight-and-a-half months pregnant? This is really crazy. But I look around and I’m like, oh, because I look exactly like all these other fat guys with beer bellies who were at this plant show, like middle-aged fat guys. That’s what we look like.*”

“*I was really pleasantly surprised by how easily people saw me as a fat man – I thought I would be really struggling to be read as male, and I wasn’t, at all*.”

Strategy 3, being out and visible (acting so as to incline others to see one as transgender), may increase internal affirmation,

“*I find that when I try and normalize myself or act normal or be more normal than I am, I become really uncomfortable and unhappy. And it doesn't help anyone. So, yeah, just sort of doing it my own way and knowing that I was doing it my own way was a really helpful strategy*.”

Being visible as trans men allowed for affirmation on three axes, namely of their gender as male, as trans, and of their pregnancy. However, some participants worried that it would expose them to more transphobic violence and discrimination, which was the main reason for employing strategy 1.

#### Prioritizing transition or pregnancy?

Among many participants, there was a tension between pursuing their reproductive goals and their transition goals. One participant deferred initiating testosterone therapy for over a decade until after child bearing because of uncertainty regarding testosterone and potential impairment of high priority reproductive prospects “*If they can’t give me better information about having babies, then I'm not going to start testosterone. So, in that way, it* [the decision to delay hormone therapy] *was easy to make, but it was difficult to accept.*” Another participant knew from childhood that he wanted to bear children, but held medical transition as a higher priority, so he initiated testosterone as soon as possible, despite believing it might impair future conception and pregnancy, stating, “*I still had a desire to have children one day, I just started testosterone because I felt it was necessary for me to socially transition that way. Having children was an issue for the future*.”

Some participants felt confident, based upon knowing the stories of other men who had given birth, that testosterone would not impair their ability to get pregnant, “*I had read* [about another trans man] *who had gotten pregnant after years on testosterone… So I was never really afraid I wouldn’t be able to*.” They chose their timing of testosterone and pregnancy independently, when they were ready for each. Some participants only began considering pregnancy after having already initiated testosterone.

##### Sequencing of transition relative to pregnancy

There was a diversity in how participants sequenced pregnancy and transition.

##### Social transition

Some become pregnant before transitioning socially, some while they were living part-time as male, and some had been living as male for over a decade before becoming pregnant.

##### Testosterone

Some participants became pregnant without having previously taken testosterone, and some had been taking testosterone and stopped taking it in order to become pregnant. Of those who had not taken testosterone before pregnancy, some had started to take it afterward, some intended to start but had not yet, and some did not intent to start.

##### Genital surgery

None of the participants had genital surgery prior to their pregnancies. Some had genital or reproductive organ surgery (metoidioplasty, phalloplasty, and hysterectomy) after pregnancy.

##### Chest surgery

Some participants had had no chest surgery prior to their pregnancies. Others had had chest reduction, with a mix of surgical techniques. Of those without prior chest surgery, some chose to nurse their child “*I fed both my kids mammal-style until they were one*,” and some did not. Of those with prior chest surgery, some produced sufficient milk and fed their child for over 6 months, some swelled but did not lactate, and some experienced no swelling or lactation.

### Structural barriers, erasure, and transphobia

Participants described myriad challenges and barriers to care throughout their process of reproductive planning, conception, pregnancy, delivery, and the postpartum period. Most of these barriers can be attributed to erasure and/or transphobia.

#### ‘Pregnant man’ as unintelligible

One pervasive way erasure functioned to disempower participants was to produce a discourse in which the notion of a pregnant man was unintelligible. “*They could not make sense of the concept at that time of being male and pregnant.*” For participants themselves, the absence of any models of transgender men choosing pregnancy was profoundly disempowering, “*that was the thing that I most wanted, was to be aware that some other people were doing it*.” Those with even one example cited it as immensely affirming of their choices and experiences. “*I had seen a documentary where a trans guy was pregnant… so that helped me roll with it when I did get pregnant… by accident.*”

#### Lack of biomedical information and provider training

There is a dearth of biomedical research and education on the lives and issues of concern to transgender people in general. This applies even more so to issues of reproduction. Participants described frustration with the lack of information on the short-term and long-term effects of testosterone on reproductive organs, ease of conception, pregnancy outcomes, mental health, and lactation. These pervasive questions directly disempowered patients through limiting information useful in informed decision-making. For example, the above participant delayed childbearing for a decade while waiting for information about the effects of testosterone. This lack of information was experienced as coming from paltry research and/or inadequate provider training. One participant articulated the importance of providers “*differentiat*[ing] *between ‘I don’t know’ and ‘science doesn’t know’*.”

Per participant perceptions, this lack of information also interacts with individual providers values. Some participants perceived women’s health providers as unwilling to treat transgender male patients.

“*I had heard many times over that* [providers] *felt uncomfortable with me. And just as a blank statement, I can only read into what that means. But they also said that they didn’t have anything to refer to.* [A transgender male patient seeking pregnancy] *was too new and too different for them, and they didn’t have studies to look at. They didn’t know if this was safe, none of that. So I think that they were afraid of helping, and getting it wrong, in addition to feeling uncomfortable.*”

This participant perceived the provider’s decision (choosing not to provide care) as the consequence of both inadequate information and personal discomfort, where neither alone would necessarily have led to that decision.

#### Lack of cultural competency

Participants reported a long list of ways that providers and medical staff demonstrated a lack of cultural competence in their interactions. Prime examples of mistreating patients due to lack of cultural competency included:

- Addressing the patient with the wrong title or pronoun, “*this one* [clinic]*, it was always ‘miss’ this and ‘her’ that.*”
- Calling the patient by their legal name rather than the name they use, “*she called me by my legal name, which is not the name I use.*”
- Presuming to know the shape of a patient’s genitals by their name or face,
- Ignoring intake forms that ask patients’ gender, “*they even asked gender and preferred name on their intake form, but the person who called me back, and the doctor, never looked at it.*”
- Presuming that a patient has, or should have, a given relationship with their body “*This midwife… forced me to reach inside and touch my babies head, even though I clearly didn’t want to*.”
- And discussing gender identity as though it is sexual orientation.

Participants described comments that were probably intended to be affirming or positive, but had the effect of tokenizing or objectifying them, “*many people said ‘Oh, you’re so amazing…’* [they] *were really trying to be kind and reach out to me. I just felt kind of tokenized.*” An example is being told, “*you should be on Oprah*,” by a nurse in the middle of an intimate procedure.

#### Transphobia

Participants describe “*getting laughed at*” by providers and nurses, having providers “*make references…to bad fiction… about trans women*,” and nurses refusing to see them. One patient described a fertility specialist who “*just thought I was too masculine to get pregnant*.” Another was denied lactation coaching in the hospital.

Participants described such events as “*transphobic*.” Recounted experiences centered around rudeness, which was more or less overt. Consider this description of a physician conveying a new diagnosis of a medically urgent ectopic pregnancy and the subsequent treatment steps:

“*It’s in the way he talks to you. It’s in the things that he says. It’s in the things that he doesn’t say. And I could tell that this physician was creeped out by me. He didn’t need to say it.*”

In addition to rudeness, participants experienced a pathologization of being transgender. For participants, this came across when being transgender was seen as a problem. Several participants reported social services threatening or attempting to remove their children from their care, even before the birth and in one case lasting years afterward.

“*Social Services* [said] *‘we’re deeming you as a risk to your child, and we’re going to try and get a court order to take her off you on the basis of neglect’.*”

#### Inappropriate medical care

Patients reported that some providers performed seemingly unnecessary physical exams – especially pelvic exams – and asked questions that felt prurient, exotifying, voyeuristic, and superfluous to the patient’s care. “*The doctor asked me some weird questions that didn’t have to do with the* [reason I was there]*, but with my* [genitals].”

One participant described an example of how the specter of transphobia can act as a barrier to appropriate care:

“*I had a wound on my finger that did not heal for 6 months, while I was breastfeeding… The first doctor I told that I was trans, and that I was breastfeeding, and that I could not take any medicine that would harm the child. Then he asked me some weird questions that didn’t have to do with the wound, but with my being trans and breastfeeding a baby. So I went to another doctor. I did not tell I him was trans, so I did not tell* [him] *that I breastfed… I got some medicine, which evidently goes into the milk and would harm the baby. So I tried to take these medicines. The baby got sick. I stopped taking the medicines, and I decided to go to a third doctor…* [In all,] *I went to see five different* [doctors]*. This is why I hardly ever go to see a doctor now.*”

In this case we see a patient receiving what they see as inappropriate medical care due to provider unfamiliarity with the patient’s obstetric history and current breastfeeding. In attempting to access appropriate and comfortable care, the patient incurred an extra burden in time and resources. Moreover, the patient’s repeated experiences with care that they perceived to be culturally inappropriate served as a deterrent to the patient seeking further care.

Another participant said, “*I never really wanted to do a home birth… I was only going to have a home birth just out of fear of how the hospital wouldn’t be able to deal with me.*” Here, we see a patient avoiding the hospital and changing care potentially for less support, during a medically intense time – labor – out of fear of transphobia, discrimination, and invasive experiences.

Some participants were denied reproductive care because of provider attitudes about their gender,

“*I went to this doctor… to sign the form to get donor sperm…and he made me see the clinic psychologist to gauge whether or not I’d be fit as a parent. And so she saw me and* [my spouse]*. And then after that it went to their ethics board, and the ethics board said that they weren’t going to treat us. So* [the doctor] *turned us away.*”

Other participants who live their lives as ‘out’ men discussed how they pretended to be women in order to avoid such barriers at sperm banks and clinics. As a private service, sperm banks are allowed to determine whether or not to provide sperm to any given client, based upon that bank’s judgment and many require prior medical approval from a physician [15]. These participants perceived, through personal experience, ‘word of mouth,’ or general caution that sperm banks are likely to deny sperm to a client who does not meet their norms for prospective parents. Some clients opted to reduce their risk by “*let*[ting] *them think I was female*” and “*I didn’t want to risk a problem, when I could avoid it, and the stakes were my ability to get pregnant…*”

Some transgender men even experienced barriers to care from providers who provide gender-affirming care (i.e., hormones and surgery), stating that they had to conceal their reproductive goals in order to receive appropriate gender-related care. One participant noted: “*Then they would definitely think you are not really trans if you still want to have a baby,* [and] *so they would not* [give you hormones]*.*” Here, participants perceived that providers’ norms (i.e., that only women choose to become pregnant) would lead providers to deny care to transgender men because of their reproductive intent.

#### Institutional erasure

“*But mostly just don’t make assumptions. That’s the main thing, if you would just not make assumptions. And I guess that sounds kind of weird to probably a lot of people who treat pregnant women. Because they’re like, what do you mean? If someone’s pregnant, then they must be a woman. I’m like, no, that’s actually not true. So I think like if you could get people to grasp that, then you’d have made a lot of progress.*”

Many OB/GYN spaces “*feel like they only cater to women giving birth*…*and that made me feel alienated.*” This was true in the physical space and decoration as well as education materials with mottos, pamphlets, posters, etc. Many participants noted that they had challenges even with physical space wherein clinics only had restrooms for women. This is in keeping with other literature on the topic [3, 16, 17].

Participants described information systems that did not have the capacity to account for a man needing services traditionally ascribed to female-only patients, in several ways. First, men who needed obstetrical (e.g., prenatal or post-partum care) or gynecological services (e.g., pap smears, cervical sexually transmitted infection testing) often faced challenges with booking or billing for those services, because of how computer and filing systems were managed. Second, many record systems did not have the capacity to differentiate between a patient’s legal name and the name they should be called. Third, although some clinics had intake forms on which patients could accurately report their gender, participants reported that many providers did not refer to these forms during visits. Finally, most men in this study reported that it was difficult or impossible to be listed as ‘father’ on their child’s birth certificate, despite this being their parental identity. Some had to undertake a legal battle, or even adopt their own children, in order to be legally recognized as a father. Overall, participants felt that these combined conditions conveyed the message that their lives could not exist within the system, and their identities did not matter.

### Positive experiences with healthcare providers

While many participants experienced mistreatment throughout the healthcare system, many also reported having positive healthcare experiences. Positive experiences were characterized both by the presence of positive features in clinical encounters (e.g., privacy, gender affirmation, and normalization) and by the absence of aversive features (e.g., misgendering, invasive questions, or exotification).

Participants consistently described the use of their appropriate name and pronouns as fundamental to feeling safe. A few patients described whole healthcare teams who were consistently good about this,

“*And they were, like, super-conscientious about it. Like we were off on the side where people wouldn't be barging in. And they were consulting me before anybody came in the room. And they were using the right pronouns. And they were not weird about it. They didn't ask me any weird questions. It was just unbelievable. I was just kind of blown away at how good they were about it.*”

This participant perceived their treatment to be exceptional and described this in direct contrast to their more common experiences with care that was much less gender affirming.

Naming and normalizing the patient’s gender can be valuable, if it can be done genuinely:

“*I walked in and the doctor who I saw, like, the very first one, she was, like, ‘Look, you're not the first pregnant guy we've had. So don't worry about that…’ I just really prefer if healthcare providers can act as though it's not exceptional or weird to be trans.*”

Additional safety seemed to come from explicitly not identifying transgender experiences as exotic or medically unique, as one participant noted:

“*I … really dislike it when people are like, ‘So, that must be so interesting to be trans’.*”

Participants spoke highly of providers who responded well to being outside familiar territory, either medically or culturally, “*She took it upon herself to educate herself, … and learned what she could before my next visit.*” They appreciated when providers did not expect their patients to teach them, but listened and learned when the patient did teach. Providers built trust by differentiating between what they themselves did not know and what medicine in general did not know. When providers could not find satisfying evidence-based guidance – such as whether it is safe to start taking testosterone while still breastfeeding – some providers were good at discussing the uncertainty with their patient, and jointly evaluating risks. This is in contrast to other providers whose style of approach seemed to be that of reflexively ruling out any approach that had a hypothetical risk.

One participant observed a common feature of providers with whom he had good interactions. “[They are] *appreciative of the fact that their regular day-to-day routine is shaken up a little bit. As opposed to freaked out.*”

Many reported having had one provider (often a primary care provider or obstetrician) with whom they had a good relationship. These same participants continued to note difficulty with other providers, such that having a good provider did not attenuate other experiences but did delineate between some positive and negative patterns of interactions in the ways they were treated.

A common theme was the participants’ difficulty in identifying in advance a provider with whom they could have a positive relationship. Some successfully found good providers through community networks or health organizations specifically serving the lesbian, gay, bisexual, and transgender communities. Some happened upon a provider who was initially not well informed, but who was able to build a good relationship and pursue guidance on how to provide medically and culturally appropriate care. However, some struggled to ever find providers with whom they felt safe. On the whole, although participants wanted their providers to be able to answer all their biomedical questions about transgender-specific situations, what they cared about more was being accepted and respected for who they were.

### Anticipatory guidance throughout the family planning process

Participants described a number of ways in which they were surprised by their experience, and frequently offered advice or information they wish they had received early in their process. One common theme was that, when patients are seeking care for transition (hormones or surgery), their providers should initiate discussions about reproductive options. Participants wished they had had better information about fertility preservation (e.g., egg cryopreservation or embryo preservation) early in their decision-making. They also wanted information on the impact of gender-affirming procedures (both medical and surgical) on future reproductive health and function (e.g., the effects of chest reconstruction on lactation, the effects of testosterone on future fertility, and the ability to carry a pregnancy). Patients wanted a general description of options and known and unknown impact of these procedures but also wanted to understand the specific logistics around fertility preservation procedures. They also stressed that this information should not only come from reproductive health providers but from those who were initiating and/or facilitating gender-affirming procedures. For example, “*The egg freezing, the embryo freezing, it has to come from the transition providers.*” “*I wish they had talked to me about what to do if I wanted to get pregnant, when they gave me T* [testosterone]*.*”

Another common theme was unanticipated emotional experiences associated with stopping testosterone, being pregnant, and/or the postpartum period. For some, these shifts in emotions were entirely unanticipated, and others still found them challenging even if they suspected they might occur. Many participants experienced a stable mood throughout the processes of discontinuing testosterone, being pregnant, and the postpartum period. Some described a very positive experience, “*being pregnant… I just felt great.*” Some of the participants who had been on testosterone reported struggling with emotional changes after stopping testosterone, while pregnant, and/or in the postpartum period.

“*Healthcare professionals need to know that postpartum depression needs to be talked about more, and it really needs to be talked about with trans men who plan on having babies and plan on breastfeeding, meaning that they won’t be getting back on testosterone to level out the hormones. Because that roller coaster was an insanity you cannot describe.*”

These participants wished that someone had advised them that such moods might happen. They also expressed a desire for normalization and contextualizing these moods as part of rapid hormonal changes and not a sign of some other medically concerning problem.

“*I hated being pregnant. It was just awful… The thing that helped the most… is my friend saying ‘it’s okay to hate being pregnant, it doesn’t mean you’re a bad parent….’ It helped me be okay with it…*”

While this may be true for any pregnant patient, many participants linked their prior testosterone use and their tenor of emotional experience surrounding pregnancy. Many participants had no memory of being advised about postpartum depression before giving birth, or of having discussed it with providers afterward, and felt ill-equipped to differentiate depression from less concerning mood swings.

“*By then I had seen a lot of providers, and no one had discussed postpartum* [depression] *with me. I thought it was normal until* [my family member] *told me I was sick and needed to see someone.*”

### Optimism

Participants perceived a recent rapid increase in the incidence of transgender men getting pregnant. This increasing visibility was often tied to optimism and hope, insofar as increasing familiarity with the topic among providers would make it easier for other transgender men in future pregnancies.

“*I think today it’s better because there are more people coming forward giving birth, and it’s not such a mind blower like it was when my pregnancy came up. Now that it’s out there, it’s like, yeah, we’ve seen this before. And more people are supportive.*”

“*Times are changing, and there are a lot of gay* [transgender men] *out there, some of whom are getting pregnant.*”

“*Ten years ago I probably would have been sent to a psych ward to have my baby taken away. But ten years from now I hope that things will be even better than* [they were for me]*. This really* [will] *become normalized.*”

## Discussion

### Empowerment

The act of choosing to visibly bear children as transgender people – as men – is growing in social visibility. These are acts of empowerment, in that they overcome barriers that have previously denied reproductive choices to transgender individuals. The barriers to full choice are myriad, and participants in this study overcame them in ways large and small, in choosing to bear children, and in the many ways they navigated specific barriers.

### Sequencing of surgical, medical, and social transitions relative to pregnancy

Given the range of transition goals of transgender people in general [18], the range of priorities for transition versus pregnancy, and the mix of intentionality of these pregnancies, it follows reasonably that transgender people will become pregnant at different times relative to social, medical, and surgical components of transition. Our participants reflected this diversity. Sequencing major life events such as transition and childbearing is a critical strategic choice. Participants’ agency in making different and personalized choices is an encouraging indicator of their empowerment. Providers and others aiming to facilitate the empowerment of transgender men around pregnancy, should encourage and support those individuals in whatever strategic choices they make.

While all the participants in this study eventually did socially transition, after their pregnancy if not before, some transgender individuals choose not to transition (i.e., they identify as a gender other than their assigned sex, but they continue to live as their assigned gender). It is likely that some men who give birth will not transition, so providers and others should inquire about individual patients’ goals and expectations (e.g., not assume that a male-identifying patient with female-assigned sex will choose to live as male).

It is worth noting that, while no one in our study became pregnant while taking testosterone, we cannot rule out that possibility in others. Additionally, there are reasons other than desiring pregnancy that some transgender men stop taking testosterone, such as barriers to accessing it, or fluidity of identity and goals. Therefore, a patient discontinuing testosterone should not be taken as a definitive indication of desiring conception. Such a patient should be informed that they will likely resume ovulation, and may become pregnant depending on engagement with penile-vaginal intercourse and whether contraception is being employed.

While a hysterectomy or bilateral oophorectomy (without subsequent estrogen and progesterone supplementation) would prevent any future pregnancies, other procedures used as part of gender affirmation, such as metoidioplasty, scrotoplasty, or phalloplasty, would not impede pregnancy, and further research is needed on whether they affect the prognosis for successful vaginal delivery.

Variation in participants’ ability to lactate after having had chest surgery likely reflects the different surgical approaches. Variation in a transgender man’s choice to chestfeed/breastfeed or not likely represents a range valuation of the benefits of chestfeeding, as well as the effect on the parent’s own physical, mental, and social health and capacity to lactate or not. An individual’s ability to make a meaningful strategic life choice relies on their being properly informed of the likely consequences of those choices. Providers play an essential role supporting empowered partnership-based medical decision-making in addressing the question of future lactation with their patients considering chest surgery.

If a transgender man is chestfeeding/breastfeeding (or pumping and feeding the child that milk), the provider should help them evaluate the possible risks to the child against the benefit of testosterone to the patient. Ruling out taking testosterone while nursing, regardless of the benefit to the parent, is an approach that implicitly values an unknown and possibly small effect on the child [19] over a known significant benefit for the parent. These relative risks and benefits as well as the medical uncertainty around these decisions should be presented to and discussed with patients with an eye towards global harm reduction. Table 2 presents the considerations regarding the intersection of hormonal and surgical transition with reproduction.

**Table 2:** Reproductive considerations for medical and surgical transition

When discussing transition options with patients, discuss the reproductive consequences. These are salient points to cover: Testosterone: - Testosterone should not be considered a form of contraception [1]. - Patients should avoid getting pregnant while taking Testosterone – it is considered a teratogen [5]. - Conception and pregnancy can occur after even long-term testosterone use [1]. - Testosterone likely decreases conception rate through ovarian suppression, however we can’t currently quantify the direct impact on ovulation or conception rates. - If genetically related children are desired or potentially desired in the future, consider storing oocytes or embryos prior to initiating testosterone. (Note: ovarian tissue preservation is still considered experimental) [25–29]. - Patients need to stop testosterone in order to pursue carrying a pregnancy. - If genetic children are desired after initiation of testosterone, testosterone should be stopped. The determination of whether and to what extent assisted reproductive technologies (ART) will be used will depend on the trans man’s a) desire to carry the pregnancy, b) presence of normal menstrual cycle, and c) the desired method of joining sperm and egg [25, 28, 29]. Chest surgery: - Chest feeding may be possible after certain forms of chest reconstruction [5, 30]. - It is not possible to tell prior to attempting to chest feed whether this is possible based on type of surgery, chest anatomy *etc*. - Discuss the likely impact of various surgical approaches on ability to chest feed / lactate. - Discuss methods used by transgender men to chest feed after chest reconstruction. - Encourage the patient to discuss these issues with their surgeon (ideally prior to surgery). - Encourage lactation support if desired. - If chest feeding is not possible or not desired discuss other methods for infant feeding and bonding. Genital surgery: - Metoidioplasty, scrotoplasty, or phalloplasty do not, by themselves, impair future reproductive options, but would likely necessitate a cesarean section for delivery. - Vaginectomy combined with hysterectomy and/or oophorectomy would eliminate the chance of future pregnancies. If patients might want biological children someday, they should consider storing oocytes, or embryos prior to genital surgery. Ovarian tissue preservation is still considered experimental [28]. Postpartum Testosterone: The effects of taking testosterone while lactating are unknown. There are possible risks to the child, but no clear evidence of harm. The benefits to the parent’s mental, emotional, physical and social wellbeing are likely highly variable, and best evaluated by the patient. - If a patient does resume or initiate testosterone while nursing, counsel them on how to look for signs of androgen exposure in the infant and encourage them to let their child’s pediatrician know.	

### Structural barriers, erasure, and transphobia

#### Pregnant man as unintelligible

Our findings demonstrate the widespread ways that discourse and norms shape people’s responses to pregnant men, and the importance of awareness in improving the experience of those individuals. It is very difficult to make a particular strategic life choice, such as carrying a pregnancy, when one has no examples of others doing so. As a result, one may not even be able to conceive of this potential life choice as a personally viable option even if otherwise physically, emotionally, and socially feasible. The lack of visible examples of transgender men going through pregnancy and birth may also lead providers to feel uncomfortable or ill-informed resulting in difficulty providing appropriate care.

Our findings focused on barriers to health within healthcare delivery settings, but a critical barrier to transgender individuals’ empowerment and health comes from outside of healthcare settings. Transgender people are exposed to high levels of individual and structural violence, which affects their health directly, and also affects how they may engage with medical care. This was reflected by many participants who noted the risks of physical violence against transgender people, as well as how legal discrimination leads to economic disempowerment, “*…the lawmakers who seem to be hell bent on making sure that I don’t have an appropriate life, that I can’t raise my kids, that I can’t find a job that’s willing to pay me.*” This speaks to the importance of legal and political protections for transgender individuals as part of furthering their empowerment in many areas of life, including reproduction and healthcare.

The limited scope and time spent on training about transgender health in most nursing programs, medical schools, and residency programs [20–22] creates a condition where participants feel they need to inform their providers about how to care for them. The National Transgender Discrimination Survey found that one in two transgender people needed to inform their providers on how to care for them [6]. In the absence of sufficient training, even the best-intentioned providers are likely to miss chances to provide medically and culturally appropriate care. Furthermore, less motivated providers are likely to make gross errors. More education and training is needed to improve the quality of care provided to trans patients and support their empowerment in medical settings. Table 3 provides suggested resources for furthering providers’ education and Table 4 delineates features of the local regions that providers should be prepared to identify.

**Table 3:** Resources for providers

- Guidelines on transgender men and pregnancy [5]. - Guidelines on transgender men and gynecologic care [3]. - “LGBT Gender Nonconforming and DSD Health” AAMC Video Series (at https://www.aamc.org/initiatives/diversity/learningseries/). - Fenway Institutes LGBT healthcare guidelines [2]. - Trans Bodies, Trans Selves [31]. - UCSF Center of Excellence for Transgender Health, Guidelines for the Primary and Gender-Affirming Care of Transgender and Gender Nonbinary People (at http://transhealth.ucsf.edu/trans?page=guidelines-home - Find local transgender or LGBT community centers, for trainings and referrals - Note that resources that are good for LGB patients aren’t necessarily good for transgender patients.

**Table 4:** Questions for providers

- What resources do you have available to help potential parents through all aspects of pre-conception counseling, pregnancy, birth, lactation and early child-rearing for children growing up with transgender parents? - What services are available to support assisted reproduction for transgender individuals in your area? - What are the best options in your area for patients to find a good environment for labor and delivery? Which hospitals, birth centers, and midwifery practices are most likely to provide appropriate care? How can you help link your patient with these resources, and how can you serve as an ally to help these birth sites best serve your patient? - What is the process for obtaining a birth certificate in your jurisdiction? How can you help patients to navigate this process in a way that affirms their identity?	

#### Lack of cultural competency

While a lack of familiarity with the basics of transgender experiences and poor biomedical understanding of medical transition are distinct forms of erasure, they reinforce each other. A provider who was never been taught about transgender health may be less likely to see a transgender patient as a normal, reasonable human, and a provider with limited understanding of transgender identities may be less likely to seek out information about transgender health.

Literature on microaggressions suggests that biased behaviors, which, individually, seem of minor significance, can become powerfully aversive in the felt experience of someone who experiences these behaviors repeatedly [23]. For example, an individual provider misgendering a patient may seem a small error to the provider, while for the patient it may serve as a reminder of the thousands of times their identity has been denigrated. An important component of empowerment is access to healthcare in which patients are safe from such denigration.

#### Transphobia

Our participants’ plethora of examples of mistreatment – subtle and overt – illustrate why some transgender people are distrusting and avoidant of institutional healthcare. Participants were misgendered, laughed at, and told they could not make good parents.

Most of our participants reported having to choose between either (1) concealing their identity and other medically relevant information in order to receive compassionate care, or (2) disclosing their identity and risking being subjected to invasive procedures and inappropriate questions that felt objectifying. When individuals cannot confidently exercise the option of being out and receiving appropriate care, they are disempowered. When patients face this tension, all parties are limited – providers are likely to not get all the information they need from the patient to most comprehensively serve them and patients are unlikely to get the best care from the provider.

### Positive experiences with healthcare providers

The positive experiences some participants described give reason for optimism. At the same time, the surprise that accompanied these stories highlight how much more work is needed. Appropriate care was seen as uncommon or exceptional, while patients perceived the norm as being uncomfortable, objectifying, or invasive.

Tables 5 and 6 provide recommendations on how to empower patients and enhance cultural competency within a medical practice and larger institution.

**Table 5:** Recommendations for clinic setup and intake

- Consider the name of the clinic and how it is represented broadly as who gets services there. A “women’s clinic” may not be the best title for a place that serves trans men and other gender expansive individuals. - Physical Space: o Ensure bathrooms are accessible to all. This means having non-gendered restrooms, not just male restroom and female restrooms. This may also mean having single use non-gendered restrooms. o Ensure signage, magazines and pamphlets speak to people of diverse backgrounds in terms of race/ethnicity, sexual orientation, and gender identity. - Broadly display a non-discrimination statement. Examples can be found at here (http://www.hrc.org/hei/sample-patient-non-discrimination-policies#.V0PlrVczyAY ) - Printed materials and signage: o Ensure language used in your institution’s literature, publicity, patient education materials, is welcoming to all people regardless of gender identity. Consider whether your clinical space suggests that women are the only people who get pregnant or are welcome at your clinic and take steps to rectify that. - Staff Training and Procedures: o Ensure all staff ask preferred name(s)/pronouns, document them, and use them consistently. o Consider how patient check-in procedure and clinical space may be comfortable only for individuals who are female identified. Could you make it comfortable for individuals who are male identified or trans identified as well as female identified? Consider how people's names are used and documented and communicated between members of the care team. o How is the phone answered? Teach staff to not assume gender from patient’s voice or assume patient status or not from voice. - Medical Records and booking: o Make sure you can book, document, and bill OB/GYN procedures and encounters for someone whose gender signifier (in your system and/or the insurance records) is male.

**Table 6:** Recommendations for clinical encounters

- Reflect the language patients use to describe their reproductive organs and bodies (e.g., chest feeding rather than breast feeding; or “front hole” instead of vagina) - Plan to educate yourself, rather than relying upon your patient to teach you. o See table on resources - Be open to your patients’ expertise and learning when they want to share. - Explain why sensitive questions are relevant; ensure these questions are clinically meaningful and not motivated by idle curiosity. - Continue to maintain good medical care and judgment, do not attend so entirely on being gender savvy that you neglect routine protocols. Note: there is a long history of transgender people facing abuse, objectification, and neglect both within and beyond healthcare settings; this may frame your encounters.	

### Anticipatory guidance throughout the family planning process

Findings suggest a persistent theme of informational isolation. Participants reported having to navigate many unknowns through informal networks and received little guidance from medical providers. Anticipatory guidance and affirmative normalization may empower patients through improving their relationships with their own process, supporting informed decision-making and improving patient-provider relationships.

Tables 7 and 8 outline the recommended topics to address with patients.

**Table 7:** Recommendations for normalization

- Encourage provider and staff comfort with the prospect of male and masculine patients being pregnant and giving birth. - Explicitly affirm transgender patients’ reproductive choices. o It may help some patients improve their relationship with their own experience. o It may improve the patient-provider relationship. - Specific points around which to enhance provider comfort and encourage normalization, include: o The desire to be pregnant o Choosing pregnancy before, concurrent with, or after transitioning medically, surgically, or socially o The choice to continue or terminate a pregnancy o The range of emotions patients may experience throughout the process of family creation o The choices parents make about how to feed their infants

**Table 8:** Recommendations regarding emotions and hormones

- Some men have significant shifts in their emotions when they stop taking testosterone, are pregnant, and during the postpartum period. This may be especially likely if they have been on testosterone previously. - These changes may be felt as positive or as negative. - Advise patients, at all stages, that they may experience such changes, and that if they do: o They should seek help from you or others if in distress or at risk of hurting themselves or others. o The quality of their emotional experience does not reflect upon their gender or the appropriateness of their pregnancy. - Monitoring: be vigilant for post-partum depression, and discuss it with patients – it may be exacerbated or altered by the patient’s experience with hormones.	

### Optimism

Participants described a perception that the incidence of transgender people giving birth is increasing, and an optimism that this will bring about improving circumstances for transgender individuals experiencing pregnancy in the future. This perception could be strictly a change in visibility, but likely represents a true shift, perhaps driven by cultural changes making non-binary transition more legible, increasing legibility of being pregnant and male, and possibly by increasing numbers of people transitioning younger in life. This possible increase in incidence, combined with increasing visibility of transgender individuals nationally, may have us poised at the brink of a sea change in how healthcare providers and institutions care for their transgender obstetric patients. It is the considered hope of the authors that increasing awareness of the experience of yesterday’s transgender patients will further the empowerment of tomorrow’s.

### Strengths and limitations

This study was able to recruit enough members of an elusive population to meet conceptual saturation. We described a wide range of experiences in this population, with significant depth and texture. These findings are based upon the experiences of those who have themselves experienced pregnancy while male, giving voice to a topic not well represented in the literature.

Nevertheless, this study was limited to English speakers, and participants were entirely from the US (80%) or Western Europe. Furthermore, because of the original study inclusion criteria, the present study excluded transgender and gender-nonconforming individuals who did not identify as male, as well as those who did not have pregnancies resulting in live birth. We are also missing the experiences of transgender men who chose to never conceive, those who wished to conceive but were not able to, or those who conceived but whose pregnancies ended in miscarriage, abortion, or stillbirth. As other studies have shown, participants engaging in online studies are disproportionately educated and economically secure [24]. Additionally, since advertisements for the study were distributed through transgender websites and community centers, our sample may not represent those who are not connected to these services.

### Future research

Participants in this study expressed some clear values on research priorities, in particular a consistent aversion to research that sees their existence as something that needs to be explained or justified, e.g., “*I’m not interested in ‘why some people are born trans and some people are not’*” and “*We do not need to ask the questions, ‘how do you dare to become pregnant, although you are a man?’*”

Research is needed on the consequences of transition-related procedures (including testosterone and surgery) on future fertility, pregnancy, child health, and lactation. Additionally, understanding how transgender men who had given birth were navigating social relationships with their communities and their children is as of yet almost entirely unexplored. These investigations will help support making society and healthcare more safe and accepting for them.

Future research on this topic should attempt to more fully represent the experiences of individuals who have lower incomes, have completed less formal education, and are racial/ethnic minorities, as well as those who have not given birth, for a variety of reasons. Investigations into the pedagogy and efficacy of various training programs will help enable future advocates to better improve obstetric healthcare for transgender and gender nonconforming individuals.

## Conclusions

The primary set of findings of this study is the range of experiences and needs of patients. The first, and most central, element is that some of the people who need obstetric care are not women. Our findings revealed broad diversity in the experiences, circumstances, and degrees of empowerment of men who are pregnant and give birth. This study represents an illustration of the diversity of experiences and serves to familiarize readers with examples of what they may encounter. These findings should guide providers on what questions to consider, more than providing definitive information about any given transgender patient.

Given the rarity of truly adequate care reported by participants, providers should actively work to ensure that their teams and institutions are comfortable and competent in working with transgender patients. Conclusions regarding guidance to providers are presented.

## Additional file

Additional file 1: Open peer review. (PDF 104 kb)
