# Supplementary material for: From erasure to opportunity: a qualitative study of the experiences of transgender men around pregnancy and recommendations for providers
Source: BMC Pregnancy Childbirth. 2017 Nov 8;17(Suppl 2):332. doi: 10.1186/s12884-017-1491-5 (PMC5688401; doi:10.1186/s12884-017-1491-5)
Supplement: Additional file 1: — Open peer review. (PDF 104 kb) [file 12884_2017_1491_MOESM1_ESM.pdf]

Reviewer reports

Title: From erasure to opportunity: a qualitative study of the experiences of transgender men around pregnancy and recommendations for providers

Supplement Managing Editor: Ushma Upadhyay
The introduction seems to address themes of empowerment in the introduction, but the discussion and conclusion fail to address the theme of empowerment. Because this Special Issue is on the theme empowerment, we would like you to strengthen the connections to empowerment. It would be helpful if the authors could include a definition of empowerment – and then make the theoretical link of how this paper will address trans men's empowerment around pregnancy. I think the most obvious way to weave the empowerment theme is in the way this paper examines ways trans men can be more empowered when carrying their pregnancies to term. There are a lot of specific resources/tables referenced in the discussion and these do seem to have intention of empowering trans men.

Another question, for those who reported unintended pregnancy, did the researchers ask about whether the participants had considered abortion?

Reviewer 1: Rana Jaleel

This article offers a qualitative account of some of the needs of transmen who have given birth. The article is important as much for its concrete recommendations of how to improve transmen's reproductive healthcare as for its nuanced portrait of transmen. While the sample set is small, the diversity of experience among even this subset of transmen bears noting and repeating in the development of future research and policy. This article provides clear and needed guidance in that respect.

Is it essential that this manuscript be seen by an expert statistician?
Perhaps, but I do not feel adequately qualified to assess the statistics.

Level of interest - An article of importance in its field

Quality of written English - Acceptable.

I declare that I have no competing interests.

Reviewer 2: Deborah Mindry

From erasure to opportunity; experiences of transgender men around pregnancy, and recommendations for providers.

The authors' clearly define the problem under investigation: the challenges (pre-natal, pregnancy, and postpartum) faced by transgender men who become pregnant. This is a very under researched issue that requires much more research. The authors' lay out key areas for future research and provide very useful tables for both providers and for

transgender clients on needed resources, protocols, etc. This paper opens up a critically important area of reproductive health care research and service provision.

The methodology is appropriate for early stage research and well described. Ordinarily the research sample size is very small but is understandable and in order given the challenges in conducting this path breaking work. Though authors claim that they were able to achieve saturation within their particular sample, I would recommend that future research samples should strive to attain a sample size of at least 25-30 individual participants for qualitative research (see Shari L. Dworkin, 2012, Sample Size Policy for Qualitative Studies Using In-Depth Interviews, Arch Sex Behav, DOI: 10.1007/s10508-012-0016-6). The authors' reporting of data in the results section is appropriate and provides a rich illustration of the range of issues faced by pregnant and parenting transgender men. The quotations used support the claims made in the paper. The authors' provide a nuanced and balanced discussion of the issues faced by their participants. They provide useful recommendations for provision of care going forward as well as for future research. They chart a path and provide recommendations and resources that should be very useful to providers and transgender men given the current state of reproductive and gender transition care for transgender men.

The greatest limitation in this study, as acknowledged by the authors, is the sampling – it would be important in future work to find ways to access transgender men who are pregnant or who may desire pregnancy in the future who also come from a range of economic and ethnic and racial backgrounds. That said, this research provides an important entry point into this area of research and these limitations should not impact the decision to publish this groundbreaking research.

There are no major or discretionary revisions suggested.

The paper is well written but there are some minor revisions required prior to publication. These include:
p.14, line 299: "For example, the above participant to delayed childbearing for a decade…"
p.17, line 356: "Several participants report social services threatening or attempting to remove…" This should be in past tense in accordance with the tense used elsewhere in this section of the paper.
p. 17, line 373: "*I did not tell I him was trans,…*"
P.17, line 377: "*This is why I hardy ever…*" Should read: *"This is why I hardly ever…"*
p.26, line 591: "and further research is needed on whether they effect the…" The sue of "effect" is incorrect, it should be "affect".
p.29, line 677: "See Tables 7 and 8 for recommended topics address with patients." Should be "…topics to address with…"

I declare that I have no competing interests.

Response to reviewers

We thank the reviewers for their very helpful insights, comments, and questions. We feel that responding to their feedback has significantly improved the manuscript.
Point-by-point responses to their comments and questions follow in green text for ease of identification.

Managing Editor: Ushma Upadhyay
1) The introduction seems to address themes of empowerment in the introduction, but the discussion and conclusion fail to address the theme of empowerment. Because this Special Issue is on the theme empowerment, we would like you to strengthen the connections to empowerment. It would be helpful if the authors could include a definition of empowerment – and then make the theoretical link of how this paper will address trans men's empowerment around pregnancy. I think the most obvious way to weave the empowerment theme is in the way this paper examines ways trans men can be more empowered when carrying their pregnancies to term. There are a lot of specific resources/tables referenced in the discussion and these do seem to have intention of empowering trans men.

Text was added throughout the Background, Discussion, and Conclusion sections more explicitly discussing the significance of the research in terms of empowerment.

2) Another question, for those who reported unintended pregnancy, did the researchers ask about whether the participants had considered abortion?

A sentence was added in the 'reproductive intent' results section addressing this question.

Reviewer: Rana Jaleel

Is it essential that this manuscript be seen by an expert statistician?
Perhaps, but I do not feel adequately qualified to assess the statistics.

This manuscript does not contain statistical

analyses.

Reviewer: Deborah Mindry

There are no major or discretionary revisions suggested.

The paper is well written but there are some minor revisions required prior to publication. These include:

1) p.14, line 299: "For example, the above participant to delayed childbearing for a decade…"

Edited to remove 'to.'

2) p.17, line 356: "Several participants report social services threatening or attempting to remove…" This should be in past tense in accordance with the tense used elsewhere in this section of the paper.

Edited as suggested.

3) p. 17, line 373: "*I did not tell I him was trans,…*"

Edited to make quote more fluent.

4) P.17, line 377: "*This is why I hardy ever…*" Should read: *"This is why I hardly ever…"*

Edited as suggested.

5) p.26, line 591: "and further research is needed on whether they effect the…" The sue of "effect" is incorrect, it should be "affect".

Edited as suggested.

6) p.29, line 677: "See Tables 7 and 8 for recommended topics address with patients." Should be "…topics to address with…"

Edited as suggested.
